# Photodynamic therapy with mTHPC and polyethylene glycol-derived mTHPC: a comparative study on human tumour xenografts

**DOI:** 10.1038/sj.bjc.6690170

**Published:** 1999-03

**Authors:** H-B Ris, T Krueger, A Giger, C K Lim, J C M Stewart, U Althaus, H J Altermatt

**Affiliations:** Departments of Thoracic and Cardiovascular Surgery, University of Berne, CH-3010 Berne, Switzerland; Departments of Pathology, University of Berne, CH-3010 Bern, Switzerland; MRC Toxicology Unit, Leicester, UK; Scotia Pharmaceuticals Ltd, Guildford, Surrey, UK; Department of Chemistry, Queen Mary and Westfield College, London, UK

**Keywords:** photodynamic therapy, mTHPC, MD-mTHPC, xenografts

## Abstract

The photosensitizing properties of m-tetrahydroxyphenylchlorin (mTHPC) and polyethylene glycol-derivatized mTHPC (pegylated mTHPC) were compared in nude mice bearing human malignant mesothelioma, squamous cell carcinoma and adenocarcinoma xenografts. Laser light (20 J/cm^2^) at 652 nm was delivered to the tumour (surface irradiance) and to an equal-sized area of the hind leg of the animals after i.p. administration of 0.1 mg/kg body weight mTHPC and an equimolar dose of pegylated mTHPC, respectively. The extent of tumour necrosis and normal tissue injury was assessed by histology. Both mTHPC and pegylated mTHPC catalyse photosensitized necrosis in mesothelioma xenografts at drug-light intervals of 1–4 days. The onset of action of pegylated mTHPC seemed slower but significantly exceeds that of mTHPC by days 3 and 4 with the greatest difference being noted at day 4. Pegylated mTHPC also induced significantly larger photonecrosis than mTHPC in squamous cell xenografts but not in adenocarcinoma at day 4, where mTHPC showed greatest activity. The degree of necrosis induced by pegylated mTHPC was the same for all three xenografts. mTHPC led to necrosis of skin and underlying muscle at a drug-light interval of 1 day but minor histological changes only at drug-light intervals from 2–4 days. In contrast, pegylated mTHPC did not result in histologically detectable changes in normal tissues under the same treatment conditions at any drug-light interval assessed. In this study, pegylated mTHPC had advantages as a photosensitizer compared to mTHPC.

Tissue concentrations of mTHPC and pegylated mTHPC were measured by high-performance liquid chromatography in non-irradiated animals 4 days after administration. There was no significant difference in tumour uptake between the two sensitizers in mesothelioma, adenocarcinoma and squamous cell carcinoma xenografts. Tissue concentration measurements were of limited use for predicting photosensitization in this model. © 1999 Cancer Research Campaign


*British Journal of Cancer* (1999) **79**(7/8), 1061–1066
© 1999 Cancer Research Campaign
Article no. bjoc.1998.0170

### Photodynamic therapy with mTHPC and polyethylene glycol-derived mTHPC: a comparative study on human tumour xenografts

**H-B Ris[1], T Krueger[1], A Giger[2], CK Lim[3], JCM Stewart[4], U Althaus[1] and HJ Altermatt[2]**

Departments of [1]Thoracic and Cardiovascular Surgery and [2]Pathology, University of Berne, CH-3010 Bern, Switzerland; [3]MRC Toxicology Unit, Leicester, UK; [4]Scotia Pharmaceuticals Ltd, Guildford, Surrey, UK; [5]Department of Chemistry, Queen Mary and Westfield College, London, UK

**Summary** The photosensitizing properties of m-tetrahydroxyphenylchlorin (mTHPC) and polyethylene glycol-derivatized mTHPC (pegylated mTHPC) were compared in nude mice bearing human malignant mesothelioma, squamous cell carcinoma and adenocarcinoma xenografts. Laser light (20 J/cm$^2$) at 652 nm was delivered to the tumour (surface irradiance) and to an equal-sized area of the hind leg of the animals after i.p. administration of 0.1 mg/kg body weight mTHPC and an equimolar dose of pegylated mTHPC, respectively. The extent of tumour necrosis and normal tissue injury was assessed by histology. Both mTHPC and pegylated mTHPC catalyse photosensitized necrosis in mesothelioma xenografts at drug-light intervals of 1–4 days. The onset of action of pegylated mTHPC seemed slower but significantly exceeds that of mTHPC by days 3 and 4 with the greatest difference being noted at day 4. Pegylated mTHPC also induced significantly larger photonecrosis than mTHPC in squamous cell xenografts but not in adenocarcinoma at day 4, where mTHPC showed greatest activity. The degree of necrosis induced by pegylated mTHPC was the same for all three xenografts. mTHPC led to necrosis of skin and underlying muscle at a drug-light interval of 1 day but minor histological changes only at drug-light intervals from 2–4 days. In contrast, pegylated mTHPC did not result in histologically detectable changes in normal tissues under the same treatment conditions at any drug-light interval assessed. In this study, pegylated mTHPC had advantages as a photosensitizer compared to mTHPC.

Tissue concentrations of mTHPC and pegylated mTHPC were measured by high-performance liquid chromatography in non-irradiated animals 4 days after administration. There was no significant difference in tumour uptake between the two sensitizers in mesothelioma, adenocarcinoma and squamous cell carcinoma xenografts. Tissue concentration measurements were of limited use for predicting photosensitization in this model.

**Keywords:** photodynamic therapy; mTHPC; MD-mTHPC; xenografts

Photodynamic therapy (PDT) is a method of destroying cancer by free radical oxidation necrosis. This is achieved by photo-activating a dye (the photosensitizing agent) in cancer tissue. This process generates oxygen species, which destroy the cell organelles. It is an aim of PDT to destroy superficially localized tumours selectively while sparing surrounding tumour-free structures. However, it is well-recognized that PDT is not yet optimized in this respect. For example, several reports have documented PDT-related injuries on normal tissues in clinical (Pass et al, 1994; Takita et al, 1994) and experimental (Pelton et al, 1992; Ris et al, 1993*a*, 1993*b*; Stewart et al, 1993; Ji et al, 1994; Tochner et al, 1994; Veenhuizen et al, 1994) settings. Thus, there have been many efforts to improve selective action; for example, by developing new photosensitizers, modulating drug-light conditions, and improving light dosimetry (Ash and Brown 1993; Levy 1994). For example, m-tetrahydroxyphenylchlorin (mTHPC), a chlorin class sensitizer, has shown better therapeutic gains and a more rapid reduction of skin photosensitization than Photofrin in comparative studies in mice (van Geel et al, 1995). It has been shown in a rodent model to accumulate in high concentrations in tumours and the reticuloendothelial system but not in muscular organs such as the heart (Alian et al, 1994). In common with hematoporphyrin derivatives (HpD), PDT with mTHPC seems to act at least partly via a type II process where singlet oxygen is generated (Ma et al, 1994). The quantum yield for photoinactivation of cells is smaller for mTHPC than for other sensitizers (Ma et al, 1994).

We have assessed intraoperative intracavitary PDT of the chest using mTHPC in patients suffering from malignant pleural mesothelioma; 0.3 mg/kg body weight mTHPC were injected i.v. and laser light (10 J/cm$^2$) at 652 nm was delivered at a drug-light interval of 2 days. This resulted in tumour necrosis up to 10 mm deep (Ris et al, 1991), and suggested that mTHPC is an efficient second-generation sensitizer for clinical application. Similar findings emerged from experimental settings of other investigators (Lofgren et al, 1994; Ma et al, 1994; van Geel et al, 1995) However, as serious PDT-related side-effects were observed in our patients, we orientated our research towards optimization of mTHPC–PDT by changing drug-light conditions in an experimental setting with nude mice bearing human malignant mesothelioma xenografts. The tumour and tumour-free tissue that served as control were treated under the same conditions and the extent of PDT-related tumour necrosis and normal tissue injury were compared in order to assess the therapeutic ratio for each drug-light parameter. We observed

*Received 16 January 1997*
*Revised 14 April 1997*
*Accepted 26 June 1997*

*Correspondence to:* H-B Ris, Klinik für Thorax-Herz und Gefässchirurgie, Inselspital, CH-3010 Bern, Switzerland that the therapeutic ratio of mTHPC–PDT was highly dependent on the drug-light interval, and that best results were obtained at an interval of 3 days (Ris et al, 1993*a*). Selectivity was further enhanced in the model by decreasing the drug dose to 0.1 mg/kg body weight while increasing the light dose to 20 J/cm$^2$, 0.2 W/cm$^2$ at drug-light intervals ranging from 2 to 4 days (Ris et al, 1993*b*). We are now exploring the effect of structural modification of the sensitizer itself to see if photosensitizing effects and therapeutic ratios can be improved still further. The photosensitizing effects of mTHPC and polyethylene glycol-derived mTHPC (pegylated mTHPC) were compared with respect to their photosensitizing properties in nude mice bearing different human tumour xenografts.

### MATERIALS AND METHODS

### Tumours and animals

Human malignant mesothelioma (Reale et al, 1987), poorly differentiated human squamous cell carcinoma of the oral cavity (Altermatt et al, 1988) and poorly differentiated human adenocarcinoma of the colon (Altermatt et al, 1988) were xenografted on BALB/c nude mice using the trocar technique. At least 6 passages on nude mice were performed for each tumour type after thawing from liquid nitrogen before treatment was initiated. The tumour volume doubling time was 300 h for mesothelioma, 113 h for squamous cell carcinoma and 135 h for adenocarcinoma xenografts. One tumour was grown subcutaneously on the right flank of each animal and treatment was initiated when the tumour reached a diameter of 10–12 mm. Animal housing included artificial light in a 12 h rhythm before and after administration of the photosensitizer.

### Drug administration m-THPC (Bonnett et al, 1989) from Scotia (Guildford, UK) was dissolved in a pharmaceutical-grade solution of 20% ethanol, 30% polyethylene glycol 400 and 50% $H_2O$ for administration. Pegylated mTHPC from Scotia is a water-soluble high molecular-weight derivative of mTHPC, prepared by covalently binding polyethylene glycol 5000, to each of the four hydroxy residuals to give tetrakis (methoxy PEG 5000) ether of 7,8-dihydro-5,10,15,20-tetrakis-(3-hydroxyphenyl)-21,23-(H)-porphyrin. Its molecular weight is approximately 20 000. It was dissolved in sterile 0.9% NaCl for administration.

mTHPC (0.1 mg/kg) and an equimolar dose of pegylated mTHPC was injected i.p. into 90 mice (45 animals for each compound), meaning that the same amount of mTHPC (the active moiety) was always compared irrespective of the molecular weight of the sensitizer.

### Light delivery

Before light delivery, the animals were anaesthetized with Avertin i.p. (Sterling Winthrop, NY, USA) and kept on a warm towel pad during treatment. Argon-pumped dye laser light of 652 nm was delivered through a quartz optical fibre containing a lens. A power track system allowed for a constant power output (Coherent Innova 200 and Dye CR 599, GMP SA, Lausanne, Switzerland). In each animal, non-contact surface irradiation was performed on the tumour (through the intact skin overlying the tumour) and on an equal-sized area of the hind leg serving as control site for normal tissue. The irradiated spots were 1.3 cm in diameter and the treated surfaces were situated perpendicular to the incident laser beam. The power at the end of the fibre was measured by a power meter calibrated for 652 nm, allowing for non-thermal power density on the irradiated surfaces of 0.2 W/cm$^2$. A dose of 20 J/cm$^2$ was delivered on each spot, the treatment time (100 s) was controlled by a time shutter.

Groups of 6 animals were assessed for each drug-light condition and each compound: *mesothelioma xenografts* were treated at drug-light intervals of 1, 2, 3 and 4 days (8 groups), *squamous cell carcinoma xenografts* at a drug-light interval of 4 days (2 groups) and *adenocarcinoma xenografts* at a drug-light interval of 4 days (2 groups).

### Assessment of tumour and normal tissue injury

Seventy-two hours after light delivery the animals were euthanized by ether overdose. The whole animals were fixed in neutral buffered formalin (10%). The tumours and control sites were cut at a right angle to the surface from the centre to the periphery, routinely processed and paraffin embedded. The extent of necrosis in the irradiated tumours was expressed as an area (measured by planimetry) rather than depth as this is more accurate for inhomogeneous necrosis seen in a nodular tumour (Ris et al, 1993*b*). A transparent grid with 1 mm spacing was placed over the histological section taken through the largest diameter of the tumour and the number of grid intersections falling within the necrotic or non-necrotic tumour area were counted with the aid of a dissecting microscope (magnification $\times 16$). This procedure was repeated three times at different angles and the median value was used for statistical analysis. Since this approach could not be applied to the layered structures of normal tissue from the control site comprising skin and muscle, the maximum depth of visible change (necrosis, leucocytic infiltration, oedema and depletion of hair follicles) was measured in the control site as described earlier (Ris et al, 1993*a*). Tumour and normal tissue damage was histologically assessed by an independent pathologist (HJA) without knowledge of the treatment performed.

### Sensitizer tissue concentration measurements

Sensitized but non-irradiated animals were asphyxiated in ether 4 days after i.p. administration of 0.1 mg/kg mTHPC and an equimolar dose of pegylated mTHPC, respectively. Three animals were assessed for each type of tumour and each compound. The tumours, free of overlying tissue with a size of 10–12 mm in diameter, were dissected and muscle and skin of the hind leg as well as lung, heart, liver, kidney, spleen and gut were harvested. The specimens were irrigated in 0.9% NaCl, frozen in liquid nitrogen and stored at –70°C. The tissue concentrations of the two compounds were measured by high-performance liquid chromatography (HPLC) as follows.

#### *mTHPC*

The tissues (100–200 mg) were homogenized in 2 ml of the homogenizing medium (8 parts methanol–DMSO 4:1 v/v containing the internal standard paratetrahydroxyphenylchlorin and 1 part water) in a Dounce homogenizer. The mixture was transferred into a clean tube and centrifuged at 2600 ***g*** for 10 min.

 © Cancer Research Campaign 1999

**Table 1** Extent of photosensitized tumour necrosis and normal tissue alterations for mTHPC[a] and pegylated mTHPC[b] on mesothelioma xenografts

| Drug-light interval (days) | Tumour-free tissue (mm ± 1 SD) mTHPC | Tumour-free tissue (mm ± 1 SD) Pegylated mTHPC | Tumour ($mm^2$ ± 1 SD) mTHPC | Tumour ($mm^2$ ± 1 SD) Pegylated mTHPC |
|---|---|---|---|---|
| 1 | 2.6 ± 0.6 | 0 | 43.4 ± 32.8 | 37.2 ± 33.1 |
| 2 | 0.1 | 0 | 38.5 ± 28.2 | 55.9 ± 32.2 |
| 3 | 0.1 | 0 | 36.9 ± 16.6 | 53.4 ± 9.6 |
| 4 | 0.1 | 0 | 39.3 ± 27.3 | 86.3 ± 25.1 |

[a]0.1 mg/kg mTHPC, light dose 20 J/$cm^2$. [b]The dose of pegylated mTHPC was equimolar to 0.1 mg/kg mTHPC; light dose 20 J/$cm^2$.

A B

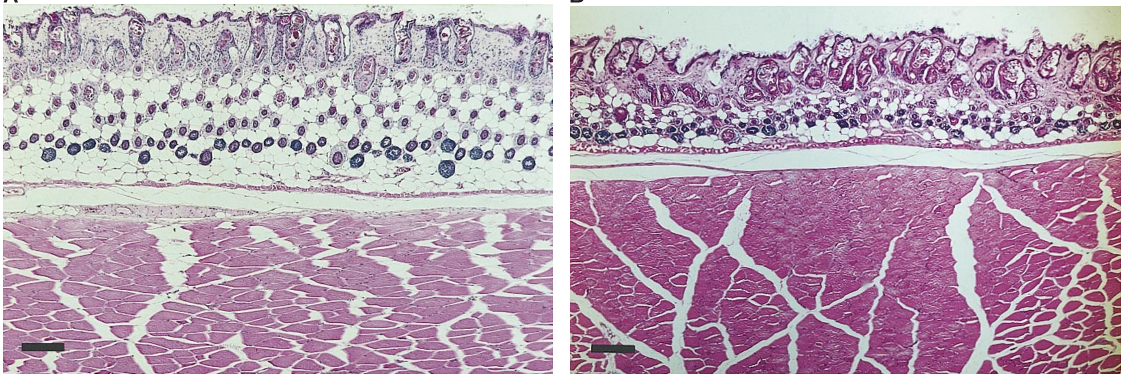

**Figure 1** Histological assessment of skin and underlying muscle of the hind leg of untreated animals (**A**), and after PDT with pegylated mTHPC equimolar dosed to 0.1 mg/kg mTHPC, 20 J/$cm^2$ and a drug-light interval of 1 day without obvious alterations except depletion of hair follicles assessment 72 h after irradiation (haematoxylin & eosin, bar = 0.2 mm), (**B**)

Four hundred microlitres of the supernatant was mixed with 200 µl of water, and 200 µl of the solution was injected. The column consisted of hypersil–ODS (5 mm) and the mobile phase of acetonitrile-0.1% TFA (77:23 v/v). The flow rate was 1 ml/min. A linear UVIS-204 detector set at 416 nm and a Perkin-Elmer (Norwalk, CT, USA) LS-3 fluorometer set at 406 nm (excitation) and 653 nm (emission) were used.

### *Pegylated mTHPC*

This was analysed as mTHPC following acid hydrolysis as follows. The tissues were homogenized with 25% HCl–DMSO (9:1 v/v) using 0.5 ml of homogenizing solution per 100 mg tissue. The homogenate was transferred to a 5 ml glass tube, flushed with N2 and heated for 1 h at 70°C. After cooling to room temperature the homogenate was centrifuged at 4000 g for 15 min. The supernatant was withdrawn and the residue extracted by vortex-mixed with 0.5 ml of MeOH/DMSO (4:1 v/v) and centrifuged at 4000 RPM for 15 min. Four hundred microlitres of the supernatant was withdrawn and the residue was mixed with 200 µl of water and again centrifuged at 8800 g for 5 min. Two hundred microlitres of the supernatant was then injected into the HPLC column.

### Controls

The histological pattern of the tumour was assessed on 6 non-sensitized, non-irradiated animals for each type of xenograft to determine the vascular architecture and the extent of spontaneous tumour necrosis.

### Statistical analysis

The Student's *t*-test for paired and unpaired observations was applied where appropriate by using a two-tailed hypothesis. Significance was accepted at $P < 0.05$.

### RESULTS

### Photosensitizing effects on tumour free tissue (Table 1)

At an interval of 1 day, substantial necrosis comprising skin and underlying muscle was observed with mTHPC as sensitizer at 0.1 mg/kg and a light dose of 20 J/$cm^2$. At drug-light intervals ranging from 2 to 4 days, mTHPC led to negligible changes (scattered leucocytes) in normal tissues using the same drug-light condition. Photoactivation with the same light conditions and an equimolar dose of pegylated mTHPC did not cause histologically recognizable changes in normal tissue at any drug-light interval assessed (Figure 1).

### Photosensitizing effects on malignant xenografts

### *Controls*

Adenocarcinoma xenografts revealed histologically a delicate tumour stroma with numerous small capillary vessels. In contrast, malignant mesothelioma and squamous cell carcinoma were poor in stroma and blood vessels. The extent of spontaneous tumour necrosis was not significantly different for the three neoplasms. It was focal-diffuse in all three types of xenografts and revealed a

© Cancer Research Campaign 1999

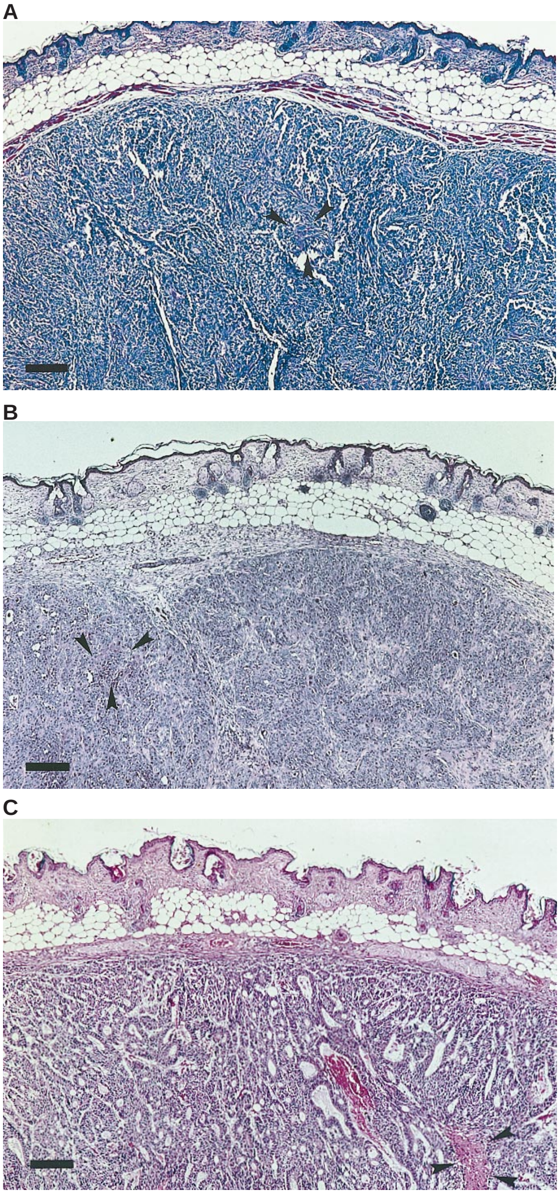

**Figure 2** Histological assessment of xenografts of non-sensitized, non-irradiated animals: malignant mesothelioma (**A**), squamous cell carcinoma (**B**) and adenocarcinoma (**C**), revealing typical small focal-diffuse areas of spontaneous necrosis (arrows) within otherwise viable tumour (haematoxylin & eosin, bar = 0.2 mm)

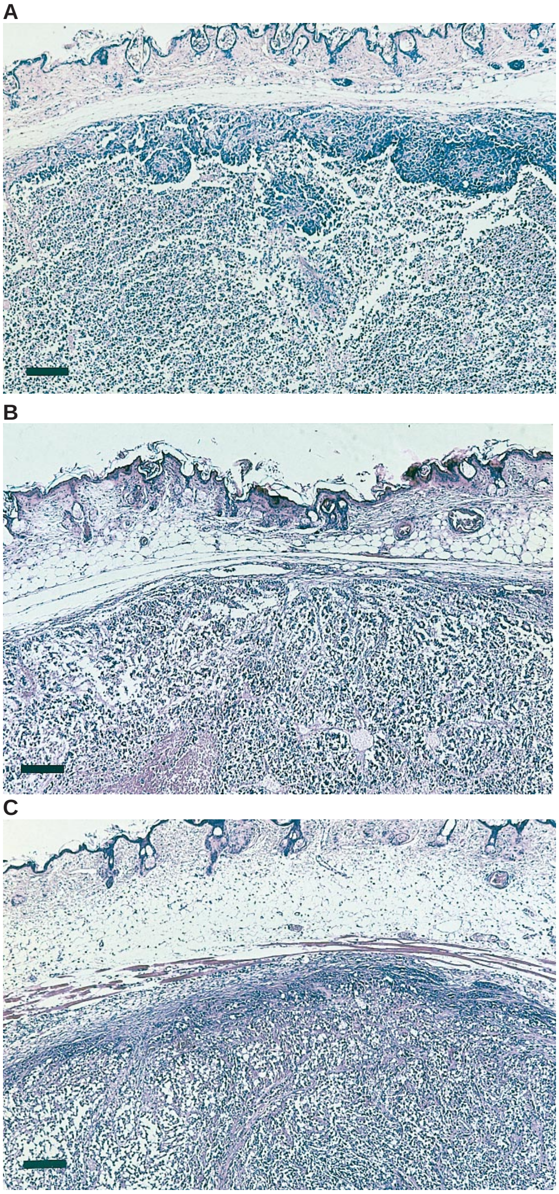

**Figure 3** Histological assessment of xenografts after PDT with pegylated mTHPC equimolar dosed to 0.1 mg/kg mTHPC, 20 J/cm$^2$ and a drug-light interval of 4 days: malignant mesothelioma (**A**), squamous cell carcinoma (**B**) and adenocarcinoma (**C**), with extensive photosensitized necrosis. Assessment 72 h after irradiation (haematoxylin & eosin, bar = 0.2 mm)

**Table 2** Extent of photosensitized tumour necrosis for mTHPC[a] and pegylated mTHPC[b] in human mesothelioma, squamous cell carcinoma and adenocarcinoma xenografts (mm$^2$ ± 1 SD)

| | Mesothelioma | Squamous cell carcinoma | Adeno-carcinoma |
|---|---|---|---|
| Control | 5.0 ± 7.2 | 6.5 ± 4.7 | 11.5 ± 7.6 |
| mTHPC | 39.3 ± 27.3 | 19.5 ± 3.8 | 83.1 ± 37.8 |
| Pegylated mTHPC | 86.3 ± 25.1 | 58.4 ± 23.3 | 81.5 ± 23.4 |

[a]0.1 mg/kg mTHPC, light dose 20 J/cm$^2$, drug-light interval 4 days. [b]The dose of pegylated mTHPC was equimolar to 0.1 mg/kg mTHPC; light dose 20 J/cm$^2$, drug-light interval 4 days.

histomorphological pattern which was distinct from that observed 72 h after PDT (Figure 2).

### *Mesothelioma xenografts (Table 1)*

mTHPC (0.1 mg/kg) and 20 J/cm$^2$ resulted in substantial PDT-induced tumour necrosis at drug-light intervals ranging from 1 to 4 days, without significant differences between these time points. Pegylated mTHPC equimolar dosed to mTHPC and 20 J/cm$^2$ resulted in a significant increase of tumour necrosis compared to that of mTHPC at drug-light intervals of 3 days ($P < 0.05$) and 4 days ($P < 0.005$). At drug-light intervals of 1 and 2 days, there was no significant difference in tumour necrosis between the two sensitizers. Comparing the pegylated mTHPC-induced tumour

© Cancer Research Campaign 1999

**Table 3** Tissue concentration measurements for mTHPC[a] and pegylated mTHPC[b] in the tumour and normal tissues (ng/g tissue, mean ± 1 SD)

| | mTHPC | Pegylated mTHPC |
|---|---|---|
| Mesothelioma | 0.07 ± 0.06 | 0.13 ± 0.08 |
| Adenocarcinoma | 0.07 ± 0.007 | 0.19 ± 0.08 |
| Squamous cell carcinoma | 0.08 ± 0.02 | 0.01 ± 0.002 |
| Heart | 0.05 ± 0.03 | 0.41 ± 0.13 |
| Lung | 0.07 ± 0.01 | 0.10 ± 0.01 |
| Liver | 0.05 ± 0.01 | 0.15 ± 0.02 |
| Spleen | 0.07 ± 0.01 | 0.16 ± 0.05 |
| Kidney | 0.11 ± 0.02 | 0.06 ± 0.01 |
| Gut | 0.18 ± 0.07 | 0.16 ± 0.05 |
| Skin | 0.07 ± 0.02 | 0.07 ± 0.03 |
| Muscle | 0.01 ± 0.003 | 0.02 ± 0.01 |

[a]Assessment 4 days after i.p. injection of 0.1 mg/kg mTHPC. [b]Assessment 4 days after i.p. injection of pegylated mTHPC equimolar dosed to 0.1 mg/kg mTHPC.

necrosis at different time points, it was significantly larger at a drug-light interval of 4 days than at 1 day ($P < 0.02$), 2 days ($P < 0.05$) and 3 days ($P < 0.01$).

### *Comparison between mesothelioma, squamous cell carcinoma and adenocarcinoma xenografts (Table 2)*

mTHPC (0.1 mg/kg) and 20 J/cm$^2$ at a drug-light interval of 4 days resulted in larger tumour necrosis than observed in controls for all tumour types ($P < 0.005$). Adenocarcinoma displayed larger necrosis than squamous cell carcinoma ($P < 0.001$) and malignant mesothelioma ($P < 0.02$), whereas no significant difference between squamous cell carcinoma and malignant mesothelioma was observed. Pegylated mTHPC equimolar dosed to mTHPC and 20 J/cm$^2$ at a drug-light interval of 4 days resulted in extensive tumour necrosis for all xenografts without significant difference between malignant mesothelioma, squamous cell carcinoma and adenocarcinoma. The extent of necrosis was significantly larger with pegylated mTHPC than with mTHPC with this drug-light condition for malignant mesothelioma ($P < 0.01$) and squamous cell carcinoma ($P < 0.001$), but not for adenocarcinoma (Figure 3).

### Tissue concentration measurements (Table 3)

There was no significant difference in the tumour tissue concentration between mTHPC and pegylated mTHPC in adenocarcinoma, mesothelioma and squamous cell carcinoma xenografts 4 days after administration of the sensitizers. No difference was found in mTHPC uptake between the three types of tumour. Pegylated mTHPC revealed a lower uptake in squamous cell carcinoma than in adenocarcinoma xenografts ($P < 0.05$).

Four days after administration, significantly higher tissue concentrations were measured for pegylated mTHPC than mTHPC in heart ($P < 0.001$), lung ($P < 0.01$), spleen ($P < 0.001$), and liver ($P < 0.001$), and a lower concentration in kidney ($P < 0.01$). No significant difference was found between the two sensitizers in this respect in gut, skin and muscle tissue.

### DISCUSSION

The ability of mTHPC to produce tumour necrosis even with small light doses under clinical (Ris et al, 1991) and experimental conditions (Lofgren et al, 1994), makes this compound attractive for its use in intraoperative PDT, given the short treatment time required. However, injuries to normal tissues have been observed in experimental settings after PDT with mTHPC, especially if high drug or light doses were applied at a short drug-light interval (Ris et al, 1993*a*, 1993*b*; Veenhuizen et al, 1994; van Geel et al, 1995). Even low doses of sensitizer (0.2 mg/kg) and light (8–12 J/cm$^2$) have resulted in severe normal tissue damage of rats after whole abdomen i.p. PDT (Veenhuizen et al, 1994), emphasizing the need for optimization of mTHPC before its routine clinical use in intra-abdominal, or intrathoracic adjunctive therapy. This study was undertaken to improve the efficacy and tumour selectivity of PDT, and compared mTHPC with pegylated mTHPC on different tumour xenografts in order to establish experimental fundamentals before clinical application.

Polyethylene glycol is a long-chain water-soluble polyether that is widely used as a pharmaceutical vehicle. Recently it has been found that covalent binding of polyethylene glycol to pharmaceuticals (pegylation) confers benefits beyond that where it is used as a vehicle alone (Burnham, 1994). These include decreased immunogenicity and absorption by the liver, increased drug circulation time and enhanced tumour uptake for anticancer drugs. So far pegylation has mainly been applied to high molecular-weight substances such as proteins, enzymes and liposomes, but the pharmaceutical benefits may possibly be conferred upon other compounds. Other studies have shown that the uptake of pegylated phthalocyanine sensitizers in animal tumours is enhanced with increased tumour-to-skin uptake ratios compared to the non-pegylated compound (Allémann et al, 1995).

Pegylated mTHPC is mTHPC covalently bound to polyethylene glycol 5000 at each of the four hydroxy groups. It is water-soluble and has an average molecular weight of 20 000. Our results suggested an enhanced uptake of pegylated mTHPC in mesothelioma and adenocarcinoma compared to mTHPC 4 days after injection, but these differences were not significant. Pegylated mTHPC gave rise to better tumour-to-skin and tumour-to-muscle uptake ratios than mTHPC in mesothelioma and adenocarcinoma but not in squamous cell carcinoma xenografts. In contrast, pegylated mTHPC revealed significantly higher concentrations compared to mTHPC in liver, spleen, heart and lung tissue at this time point. Our results suggest a difference in biodistribution of the two substances similar to that observed with pegylated and non-pegylated phthalocyanines (Allémann et al, 1995).

The effect of PDT depends on a complex matrix of conditions such as tissue concentration and localization of the photosensitizer, oxygenation of the tumours, optical properties of the target tissue and on activation wavelength, power density and other treatment conditions. An intact blood supply is needed to ensure oxygenation of target tissues. Therefore, in vivo (rather than 'in vitro') investigations are essential to assess efficacy and tissue selectivity of PDT. A simple and reproducible animal model has to be chosen due to the large number of variables to be tested. Rodents bearing subcutaneously implanted tumours are an appropriate model for comparing circulation since tumours grow well and are easily accessible to PDT. However, xenografts do not reflect the natural growth pattern of the tumours or the environment where they arise in humans. We have chosen nude mice bearing human tumour xenografts since these tumours preserve features of the original tumour (Houghton and Houghton, 1987). The extent of injury in the tumour and tumour-free tissues on the same animal treated under the same drug-light conditions is the most direct way to assess the efficacy and tumour selectivity of PDT (Berenbaum et al, 1982). Our

© Cancer Research Campaign 1999

previous study with mTHPC showed that photodynamic activity does not correlate with the absolute concentration of the sensitizer in tissue measured by HPLC and does not help to predict the photosensitizing effect (Ris et al, 1993*a*). This appears to be a common finding for other sensitizers (Gibson et al, 1994). Caution is therefore indicated in predicting the photosensitizing properties of a compound according to its tissue uptake as measured by HPLC. Pegylated mTHPC resulted in extensive photosensitized necrosis in squamous cell carcinoma xenografts at a drug-light interval of 4 days despite a low tumour uptake at this time point.

The efficacy and tumour selectivity of PDT using mTHPC is highly dependent on modulations of the drug-light conditions (Ris et al, 1993*b*). mTHPC (0.1 mg/kg) and 20 J/cm$^2$ gave little damage to normal tissues at drug-light intervals ranging from 2 to 4 days but at an interval of 1 day extensive necrosis in skin and muscle occurred. With the same treatment conditions marked necrosis in malignant mesothelioma was observed without differences between the various drug-light intervals assessed (1–4 days). The same drug-light conditions at an interval of 4 days led also to photosensitized necrosis in squamous cell carcinoma and adenocarcinoma. However, necrosis in adenocarcinoma was significantly larger than in mesothelioma and squamous cell carcinoma. This difference in the extent of photosensitized tumour necrosis could not be attributed to a difference in tumour uptake of mTHPC. Since the experimental setting was identical for the three tumour types, we speculate that this phenomenon might be related to a difference in histology and vascular architecture of the xenografts leading to differences in vascular-related photosensitizing effects and direct tumour cell injury (Peng et al, 1995). Whereas adenocarcinoma xenografts revealed histologically a delicate tumour stroma with numerous small capillary vessels, the xenografts of malignant mesothelioma and squamous cell carcinoma were poor in stroma and blood vessels.

In contrast, the photosensitizing effect of pegylated mTHPC was much less dependent on the histology of the tumours and the drug-light conditions applied. A further improvement of PDT comparing efficacy and tumour selectivity was obtained in this model by use of pegylated mTHPC instead of mTHPC. No obvious changes were observed in normal tissues with an equivalent dose of pegylated mTHPC and 20 J/cm$^2$ at any drug-light interval assessed, remarkably not even at a drug-light interval of 1 day where extensive normal tissue necrosis is seen with mTHPC. At the same time a significant increase in the extent of necrosis was found in malignant mesothelioma xenografts compared to that obtained with mTHPC at identical drug-light conditions but longer drug-light intervals with best results being observed at 4 days. Pegylated mTHPC also gave a uniform necrosis for all xenografts without significant difference between malignant mesothelioma, squamous cell carcinoma and adenocarcinoma, with larger necrosis in malignant mesothelioma and squamous cell carcinoma being observed than with mTHPC.

Our results indicated that pegylated mTHPC revealed enhanced photosensitizing properties than the non-pegylated compound, which might be related to enhanced targeting of this sensitizer on the tumour, and possibly within a specific site at the tumour cell. Tissue concentrations measured by HPLC were of little use to predict the photosensitizing effects of both, mTHPC and pegylated mTHPC in this model.

### REFERENCES

Allémann E, Brasseur N, Benrezzak O, Rousseau J, Kudrevich SV, Boyle RW, Leroux JC, Gurny R and Van Lier JE (1995) PEG-coated poly(lactic acid) nanoparticles for the delivery of hexadecafluoro zinc phthalocyanine to emt-6 mouse mammary tumours. *J Pharm Pharmacol* **47**: 382–387

Alian W, Andersson-Engels S, Svanberg K and Svanberg S (1994) Laser-induced fluorescence studies of meso-tetra (hydroxyphenyl) chlorin in malignant and normal tissues in rats. *Br J Cancer* **70**: 880–885

Altermatt HJ, Gebbers JO and Laissue JA (1988) Heavy water delays growth of human carcinoma in nude mice. *Cancer* **62**: 462–466

Ash DV and Brown SB (1993) New drugs and future developments in photodynamic therapy. *Eur J Cancer* **29A**: 1781–1783

Berenbaum MC, Bonnett R and Scourides PA (1982) In vivo biological activity of the components of haematoporphyrin derivative. *Br J Cancer* **45**: 571–581

Bonnett R and Berenbaum MC (1989) Porphyrins as sensitizers. In *Photosensitizing Compounds: Their Chemistry, Biology and Clinical Use*, Ciba Foundation Symposium 146, pp. 40–53. John Wiley: Chichester

Burnham NL (1994) Polymers for delivering peptides and proteins. *Am J Hosp Pharm* **51**: 210–218.

Gibson SL, Foster TH, Feins RH, Raubertas RF, Fallon MA and Hilf R. (1994) Effects of photodynamic therapy on xenografts of human mesothelioma and rat mammary carcinoma in nude mice. *Br J Cancer* **69**: 473–481.

Houghton JA and Houghton PJ (1987) The suitability and use of human tumor xenografts. In *Rodent Tumor Models in Experimental Cancer Therapy*, Kallman RF (ed), pp. 199–204. Pergamon Press: Oxford

Ji Y, Powers SK, Brown JT, Walstad D and Maliner L (1994) Toxicity of photodynamic therapy with photofrin in the normal rat brain. *Lasers Surg Med* **14**: 219–228

Levy JG (1994) Photosensitizers in photodynamic therapy. *Semin Oncol*, **21**: 4–10

Lofgren LA, Ronn AM, Abramson AL, Shikowitz MJ, Nouri M, Lee CJ, Bati J and Steinberg BM (1994) Photodynamic therapy using m-tetra-(hydroxyphenyl)-chlorin. An animal model. *Arch Otolaryngol Head Neck Surg* **120**: 1355–1362

Ma L, Moan J and Berg K (1994) Evaluation of a new photosensitizer, meso-tetra-hydroxyphenyl-chlorin for use in photodynamic therapy: a comparison of its photobiological properties with those of two other photosensitizers. *Int J Cancer* **57**: 883–888

Pass HI, De Laney TF, Tochner Z, Smith PE, Temeck BK, Pogrebniak HW, Kranda KC, Russo A, Friauf WF, Cole JW, Mitchell JB and Thomas G (1994) Intrapleural photodynamic therapy: results of a phase I trial. *Ann Surg Oncol* **1**: 28–37

Pelton JJ, Kovalyshin MJ and Keller SM (1992) Intrathoracic organ injury associated with photodynamic therapy. *J Thoracic Cardiovasc Surg* **103**: 1218–1223

Peng Q, Moan J, Ma LW and Wesland JM (1995) Uptake, localization and photodynamic effect of mTHPC in normal and tumor tissue of mice bearing mammary carcinoma. *Cancer Res* **55**: 2620–2626

Reale FR, Griffin TW, Compton JM, Graham S, Townes PL and Bogden A (1987) Characterization of a human malignant mesothelioma cell line (H-MESO-1): a biphasic solid and ascitic tumor model. *Cancer Res* **47**: 3199–3205

Ris HB, Altermatt HJ, Inderbitzi R, Hess R, Nachbur B, Stewart JCM, Bonnet R, Berenbaum MC and Althaus U (1991) Photodynamic therapy with chlorins for diffuse malignant mesothelioma: initial clinical results. *Br J Cancer* **64**: 1116–1120

Ris HB, Altermatt HJ, Nachbur B, Stewart JCM, Wang Q, Lim CK, Bonnet R and Althaus U (1993*a*) Effect of drug-light interval on photodynamic therapy with meta-tetrahydroxyphenylchlorin in malignant mesothelioma. *Int J Cancer* **53**: 141–146

Ris HB, Altermatt HJ, Stewart JCM, Schaffner T, Wang Q, Lim CK, Bonnet R and Althaus U (1993*b*) Photodynamic therapy with m-tetrahydroxyphenylchlorin in vivo: optimization of the therapeutic ratio. *Int J Cancer* **55**: 245–249

Stewart FA and Oussoren Y (1993) Functional and histological bladder damage in mice after photodynamic therapy: the influence of sensitiser dose and time of administration. *Br J Cancer* **68**: 673–677

Takita H, Mang TS, Loewen GM, Antkowiak JG, Raghavan D, Grajek JR and Dougherty TJ (1994) Operation and intracavitary photodynamic therapy for malignant mesothelioma: a phase II study. *Ann Thorac Surg* **58**: 995–998.

Tochner ZA, Pass HI, Smith PD, Delaney TF, Sprague M, Deluca AM, Harrington F, Thomas GF, Terrill R, Bacher JD and Russo A (1994) Intrathoracic photodynamic therapy: a canine normal tissue tolerance study and early clinical experience. *Lasers Surg Med* **14**: 118–123

Van Geel IP, Oppelaar H, Oussoren YG, Van Der Valk MA and Stewart FA (1995) Photosensitizing efficacy of mTHPC-PDT compared to Photofrin-PDT in the RIF1 mouse tumour and normal skin. *Int J Cancer* **60**: 388–394

Veenhuizen RB, Ruevekamp-Helmers MC, Helmerhorst TJ, Kenemans P, Mool WJ, Marijnissen JP and Stewart FA (1994) Intraperitoneal photodynamic therapy in the rat comparison of toxicity profiles for photofrin and mTHPC. *Int J Cancer* **59**: 830–836

*© Cancer Research Campaign 1999*
